# Significant heterogeneity in hand hygiene compliance between different physician groups

**DOI:** 10.1186/1753-6561-5-S6-P121

**Published:** 2011-06-29

**Authors:** O Yow, S Callery, M Vearncombe

**Affiliations:** 1 Infection Prevention and Control, Sunnybrook Health Sciences Centre, Toronto, Canada

## Introduction / objectives

Although hand hygiene (HH) is known to be the single most important factor to reduce healthcare acquired infections, studies of HH compliance in health care workers (HCWs) has been reported to be between 30-50%. Physician HH compliance is consistently lower than other HCWs. Whether the physician group is homogeneous with regard to HH compliance is uncertain. This study examines the HH compliance between different physician groups.

## Methods

Designated auditors were trained to perform HH audits in all inpatient and long term care units of our 1185-bed academic tertiary medical centre. The auditors used a standardized validated tool to audit HCW’s HH during their patient interactions. The physician group was separated into 3 categories according to their seniority: staff physician, resident/fellow physician and medical student. When the auditors are unsure which category a physician belongs to, they ask for verbal confirmation. The HH compliance of the three physician groups was compared.

## Results

Between Oct 2010 and Feb 2011, a total of 8337 HH opportunities were observed. 1292 (15%) opportunities were observed in the physician group: 486 opportunities were observed in staff physicians, 693 in resident/fellow physicians, and 113 in medical students. The overall average hospital compliance was 79%; overall physician group compliance was 65%. Among the physician group, staff physicians compliance was 71%, resident/fellow physicians was 61% and medical students was 67%. The difference between staff physicians and resident/fellow physicians was statistically significant.

## Conclusion

There is marked heterogeneity in HH compliance between staff physicians and resident/fellow physicians. The capability to detect such differences is important to direct compliance improvement efforts, with the goal of improving overall HH compliance.

## Disclosure of interest

None declared.

